# Photobuforin II, a fluorescent photoswitchable peptide

**DOI:** 10.1016/j.bbadva.2023.100106

**Published:** 2023-09-29

**Authors:** Cristina R. Ventura, Gregory R. Wiedman

**Affiliations:** Department of Chemistry and Biochemistry, Seton Hall University, South Orange, NJ, USA

**Keywords:** Photobuforin II, Buforin, Membrane active peptide, Peptide synthesis, Photoactive

## Abstract

Antimicrobial peptide buforin II translocates across the cell membrane and binds to DNA. Its sequence is identical to a portion of core histone protein H2A making it a highly charged peptide. Buforin II has a proline residue in the middle of its sequence that creates a helix-hinge-helix motif which has been found to play a key role in its ability to translocate across the cell membrane. To explore the structure-function relationship of this proline residue this study has replaced P11 with a meta-substituted azobenzene amino acid (Z). The resultant peptide, photobuforin II, retained the secondary structure and membrane activity of the naturally occurring peptide while gaining new spectroscopic properties. Photobuforin II can be isomerized from its *trans* to *cis* isomer upon irradiation with ultra-violet (UV) light and from its cis to trans isomer upon irradiation with visible (VL). Photobuforin II is also fluorescent with an emission peak at 390 nm. The intrinsic fluorescence of the peptide was used to determine binding to the membrane and to DNA. VL-treated photobuforin II has a 2-fold lower binding constant compared to UV-treated photobuforin and causes 11-fold more membrane leakage in 3:1 POPC:POPG vesicles. Photobuforin II provides insights into the importance of structure function relationships in membrane active peptides while also demonstrating that azobenzene can be used in certain peptide sequences to produce intrinsic fluorescence.

## Highlights for photobuforin II, a fluorescent photoswitchable peptide

• The proline in the putative helix-hinge-helix motif of buforin II was replaced with an azobenzene amino acid and retains alpha helical structure.
• The new peptide exhibits a unique fluorescence emission not seen in the native peptide.
• Photobuofrin II fluorescence emission increases when incubated with lipid membranes.
• Photobuofrin II fluorescence emission decreases when incubated with DNA.

## 1 Introduction

Buforin is an antimicrobial peptide composed of 39 amino acids that was isolated from the stomach tissue of the Asiatic toad *Bufo bufo garagrizans.* After this discovery buforin II, which is a truncated version of buforin (21-aa) was later found to be more potent. [1] The sequence of buforin II is: TRSSRAGLQFPVGRVHRLLRK. The peptide has a helix-hinge-helix structure common to other membrane active peptides such as melittin, and maculatin 1.1. Residues 5-10 of the N-terminus form an alpha-helix this is followed by a central proline residue (P11) which creates a hinge between the C-terminal helix (residues 12-21). [2]

Buforin II has a net ^+^6 charge and shares sequence homology with core histone protein H2A. The proposed mechanism of buforin II is cell translocation followed by DNA binding. [3,4] The proline hinge in buforin II has been found to be critical for its ability to cross the cell membrane. In experiments with synthetic lipid vesicles [5,6] and molecular dynamics simulations [7,8] a substitution of P11A resulted in the loss of the translocation function of buforin II. Instead, these peptides exhibit pore-forming behavior. Other experiments showed that retaining flexibility between the two helical segments of the peptide such as a P11G substitution resulted in a retention of function.

Azobenzene is a photoisomerizable molecule that has been recently employed in a variety of biological applications. [9], [10], [11], [12], [13] Azobenzene can be isomerized from its thermally stable *trans* isomer to its *cis* isomer upon excitation with UV light and switched back to its *trans* isomer upon excitation with visible light. The two isomers have overlapping but distinct UV–vis spectra. *Trans*-azobenzene has a π→π* transition at 320 nm and a n→π* transition at 440 nm while *cis*-azobenzene has spin allowed transitions at 280 and 250 nm and a n→π* transition with a stronger band at 440 nm. Isomerization from *trans* to *cis* azobenzene can therefore be indicated by the decrease in the peak at 320 nm and a simultaneous increase in the weak band at 440 nm.

The photoswitchability of azobenzene makes it a good candidate for a hinge substitute in membrane active peptides. [14] Further optimization of these peptides could lead to light activated membrane active peptides in which the membrane activity of the peptides can be controlled with light for applications in photopharmacology.

Photoisomerization and fluorescence are competitive photophysical processes. For exited state azobenzene the photoisomerization reaction is much more efficient than the slower fluorescence emission process. For this reason, azobenzene fluorescence is infrequently observed. [15], [16], [17] The first fluorescent azobenzene was discovered by Shimomura et al. who incorporated azobenzene into membrane lipids. [18] Subsequent to this discovery there have been more reports of fluorescent azobenzene derivatives. [19] These azobenzenes fluoresce due to a variety of mechanisms which include but are not limited to substituent effect, inhibition of electron transfer, and aggregation induced emission (AIE). As has been found in this study these photoswitchable peptides may also be useful for their intrinsic fluorescent properties. Other recent work by Frederic et. al demonstrated a visibly fluorescent azobenzene peptide in which an intramolecular interaction between the tryptophan residue and the azobenzene is hypothesized to be the cause of the fluorescence. [20] In this study the substitution of proline for an azobenzene amino acid in membrane active peptide buforin II resulted in a fluorescent peptide. Intrinsically fluorescent peptides are extremely valuable to biochemists because they enable the detection of the peptide without disrupting its native structure by labeling with typically large fluorescent compounds. Tryptophan fluorescence has been used to this end to study membrane binding in melittin and other membrane active peptides as well as more complex interactions within proteins. [21], [22], [23] This work describes a new strategy to induce intrinsic fluorescence in peptides that retains the overall structure and activity of the peptide.

## 2 Materials and methods

### 2.1 Synthesis of photobuforin II

A meta-substituted 9-fluorenylmethoxycarbonyl (Fmoc)-protected azobenzene amino acid was synthesized using a six-step synthesis first published by Aemissegger, et. al. [24] The structure is shown in Supplementary 1. The synthesis began with 3-nitrophenylic acid and 3-nitrobenzylamine hydrochloride as the starting material. First 3-nitrophenylic acid was protected with tert-butoxycarbonyl (Boc) using 4-(dimethylamino)pyridine (DMAP) as a catalyst. 3-Nitrobenzylamine hydrochloride was Fmoc chloride-protected using N,N-Diisopropylethylamine (DIPEA) under nitrogen. The Fmoc-protected 3-nitrobenzylamine was hydrogenated using a platinum oxide catalyst. The nitro group on the tert-butyl (3 nitrophyenyl)acetate from the first step was reduced to a hydroxylamine using zinc dust and ammonium chloride followed by re-oxidation with cold iron (III) chloride which produced the nitroso product. The azo bond was formed by combining the 9H-fluoren-9ylmethyl(3-aminobenzyl)carbamate with the tert-butyl 2-(3-nitrosophenyl)acetate in glacial acetic acid under nitrogen. The tert-butyl group was removed with trifluoracetic acid (TFA) yielding the final product, an Fmoc-protected *meta* substituted azobenzene amino acid (Z, for its one letter abbreviation). Characterization of the protected Z amino acid was confirmed by NMR Spectroscopy (Supplementary 2). Photobuforin II was synthesized using standard solid phase peptide synthesis techniques using Fmoc N-terminal protected amino acids and orthogonal protecting groups. Wang Resin was used as a solid support. For coupling a solution of O-(1H-6-Chlorobenzotriazole-1-yl)-1,1,3,3-tetramethyluronium hexafluorophosphate (HCTU) in 3 molar excess amino acids in 3 molar excess and N,N-Diisopropylethylamine (DIEA) in six molar excess of 0.1 mmol Wang resin in dimethylformamide (DMF) was used in each round. The first amino acid was coupled 4 times the first coupling for one hour and the successive three couplings for 5 min. Deprotection of the Fmoc group was performed using 20 % piperidine in DMF. Subsequent amino acid couplings were coupled for 5–10 min at room temperature. Ninhydrin tests were performed after every coupling and deprotection. Double couplings and capping steps (acetic anhydride: DIEA: Resin 20:6:1) were performed when needed. The peptide was cleaved using 95 % TFA, 2.5 % water and 2.5 % triethylsilane (TES) (v/v/v) at room temperature for 2 h. The peptide was then precipitated in cold ether, dissolved in 10 % acetic acid in water (v/v), frozen and lyophilized. The peptide was purified using 10 % acetonitrile and 90 % Water for 7.56 min followed by a gradient ending in 30 % acetonitrile over 57.34 min at a flow rate of 10 mL/min. The column used was a C18 Phenomenex Luna. ESI Mass Spectroscopy was used to characterize the peptide (Supplementary 3).

### 2.2 Photoisomerization experiment

UV–vis spectra were taken using a NanoDrop Spectrophotometer. An initial spectrum was taken then the peptide was irradiated for 10 min with a Blak-Ray UV Lamp (366 nm wavelength) 115 V, 60 Hz, 0.16 amps from UVP Inc. Upland, CA. An additional spectrum was then taken. The peptide was then able to be isomerized back using a blue LED. In addition to photoisomerization the molar absorptivity of photobuforin was calculated for each light condition. Photobuforin was weighed, dissolved in MiliQ water, then serially diluted for preparation of a five-point curve. Beer's law was used to calculate the molar extinction coefficient.

### 2.3 Determination of photostationary state (PSS) distribution

The photostationary state distribution of *cis* and *trans* photobuforin II was determined using a Shimadzu Nexera-i-LC2040c HPLC equipped with a photodiode array detector. The peptide was dissolved in water with 0.1 % FA (formic acid) (v/v) at a concentration of 729 µM. The peptide was incubated in both UV and visible light as described in the previous section. A gradient was run from 25 % to 45 % acetonitrile: 0.1 % FA in water: 0.1 % FA. (v/v). The gradient was held at 10 % for 3 min then increased to 30 % over 27 min. The isomers were separated using a Phenomenex Columbus C18 column (4.6×250 mm, 5 µm 100 Å). The peaks were then integrated at 241 nm where both isomers have almost identical extinction coefficients. These data are in Supplementary 4, 5 and 6.

### 2.4 Circular Dichroism spectroscopy

Photobuforin was dissolved in 30 % TFE (trifluoroethanol) in MiliQ water and placed in a Starna quartz cuvette with a 1 mm pathlength. An average of three scans was used. VL Photobuforin was untreated, and UV Photobuforin was treated with 366 nm UV light for 10 min then scanned in the dark. Spectra were taken from 190-265 nm. Percent alpha helicity was determined using Eq. 1:

(1) $$\%A=\frac{-{\left[\theta \right]}_{222}+3000}{39000}$$

where percent alpha helicity (%A) is equal to the negative of the molar ellipticity at 222 nm [*θ*]_222_ plus 3000 divided by 39000. [25]

### 2.5 Fluorescent spectroscopy

Fluorescent spectroscopy experiments were measured on a Horiba Fluorolog-3 spectrofluorometer in a Starna quartz cuvette with a 10 mm pathlength. 3D and 2D scans were collected. Photobuforin II was scanned for excitation from 220 to 500 nm and for excitation from 250–650 nm. A series of 2D scans were run at the following concentrations: 50, 10 and 0.5 µM to determine if the fluorescence was due to aggregation induced emission (AIE). Photobuforin II was excited at 290 nm and emission was collected between 300 and 500 nm. Buforin II exhibited minimal fluorescence upon excitation at 290 nm (Supplementary 7)

### 2.6 DNA binding assay

Duplex DNA was prepared from single strands to a histone-derived sequence. [4] Forward strand (AAATACACTTTTGGT) and reverse strand (TTTATGTGAAAACCA) were solubilized in sterile water, combined in equal molar amounts, frozen, lyophilized and resolubilized in TSE buffer (10 mM Tris, 1 mM EDTA, 50 mM NaCl, pH 8.0). The DNA was then heated to 95 °C for 5 min then allowed to come to room temperature and incubated in 4 °C refrigerator for 48 h. The concentration of photobuforin II was held constant at 15 µM and varied amounts of DNA. The peptide to DNA ratios were as follows: 0.25, 0.5, 1, 2, 4, 8, and 16. Wells were run in triplicate. The plate was excited at 290 nm and scanned for emission from 330 to 550 nm using a Tecan Spark Microplate reader. The fraction bound was determined using Eq. 2:

(2) $$Fraction\;Bound\;=\;\frac{F_{s}-\;F_{min}}{F_{max}-F_{min}}$$

where F_s_ is the fluorescence of the sample, F_min_ is the fluorescence of the buffer blank, and F_max_ is the fluorescence of the sample with no DNA. The fraction bound was then plotted on the y-axis and the concentration of DNA was plotted on the x-axis to give a binding curve. The binding constant was determined by an exponential fit using a simple exponential in Eq. 3.

(3) $$y=\left(1-\exp\left(-kx^{n}\right)\right)$$

where y is the fraction bound, *k* is the binding constant, x is the concentration, and n is an interaction coefficient. [26]

### 2.7 Vesicle binding assay

1-Palmitoyl-2-oleoyl-sn-glycero-3-phosphocholine (POPC) and 1-palmitoyl-2-oleoyl-sn-glycero-3-phosphoglycerol (POPG) in chloroform were measured out in a 3:1 ratio and combined. The mixture was then dried under an N_2_ stream and stored in the desiccator overnight. The lipids were then resuspended in TSE buffer (pH 8.0) to give a concentration of 20 mM lipids and freeze-thawed 10 times. The 3:1 POPC:POPG mixture was extruded through a 100 nm filter using an Avanti mini-extruder 10 times. The lipids were then added to a 96 well plate and serially diluted to give final concentrations of 5, 2.5, 1.25, 0.625, 0.313, and 0.156 mM lipids. Additional controls of TSE buffer alone and photobuforin II alone were also added to the plate. Using a Tecan Spark plate reader the plate was excited at 290 nm and scanned from 330 to 550 nm. The fraction bound was determined using Eq. (2). F_min_ is the fluorescence of the buffer alone, and F_max_ is the fluorescence at 5 mM 3:1 POPC:POPG.

### 2.8 Vesicle leakage assay

Liposomes were prepared using previously established protocols. [27,28] In short, POPC and POPG in chloroform were measured out in a 3:1 ratio and combined. The mixture was then dried under an N_2_ stream and stored in the desiccator overnight. The lipids were suspended in buffer consisting of 10 mM HEPES, 100 mM sodium citrate and 50 mM terbium chloride, freeze thawed 10 times using an acetone-dry ice bath for freezing and a sonication bath for thawing. The lipids were then extruded through a 100 nm filter 10 times and purified from the excess TbCl_3_ using a Sephadex G-75 column. The liposome fractions were then pooled. Assays were performed on a 96-well plate. Photobuforin II was dissolved in assay buffer composed of 150 µM dipicolinic acid (DPA), 10 mM HEPES, and 300 mM NaCl. The stock solution was then added to the plate and serially diluted to give final concentrations of 50, 25, 12.5, 6.25, and 3.125 µM. All samples were run in triplicate including positive and negative controls. The positive control contained liposomes and reduced triton X in assay buffer and the negative control contained liposomes and assay buffer alone. Liposomes with encapsulated Tb-citrate were then added to the wells and the plate was centrifuged to remove any air bubbles from mixing. The plate was excited at 270 nm and emission was recorded at 545 nm. An increase in fluorescence indicated that the terbium-citrate had leaked out of the liposomes and exchanged with the DPA on the outside resulting in the fluorescent Tb-DPA complex. Percent leakage was determined by Eq. 4:

(4) $$\%\;Leakage=\frac{F_{s}-\;F_{nc}}{F_{pc}-F_{nc}}\;\times 100\%$$

where F_s_ is the fluorescence of the sample, F_nc_ is fluorescence of the negative control and F_pc_ is fluorescence of the positive control.

## 3 Results & discussion

### 3.1 Photoisomerization and photostationary state distributions

**Fig. 1:**
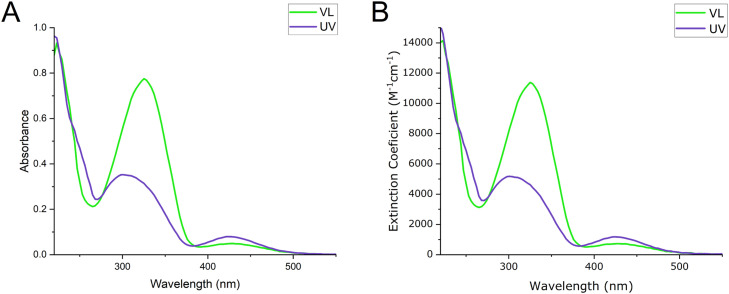
Absorbance of photobuforin II samples A) UV–vis spectra of photobuforin II and B) extinction coefficient of photobuforin II after 10 min of VL and UV light incubation.

Visible light treated photobuforin II has absorbance peaks at 330 nm and 430 nm. After irradiation with UV light the absorbance peak at 330 nm decreases and the peak at 430 nm increases. These spectral changes are characteristic of the *trans* to *cis* isomerization of azobenzene. Photobuforin II also contains a phenylalanine residue which absorbs weakly at 260 nm, but does not readily appear on the spectra due to the stronger absorbance of the azobenzene. This peak is obscured in untreated photobuforin II; however, a small shoulder peak can be observed in UV-treated photobuforin II at 253 nm (Fig. 1A). This peak may also be one of the higher intensity bands of the *cis* isomer (250 nm and 280 nm for unsubstituted azobenzene). The extinction coefficients for VL treated photobuforin II are 10000 M^−1^ cm^−1^ for the peak at 330 nm, and 700 M^−1^ cm^−1^ for the peak at 430 nm. The extinction coefficients for UV treated photobuforin II are 5000 M^−1^ cm^−1^ for the peak at 330 nm and 1000 M^−1^ cm^−1^ for the peak at 430 nm. (Fig. 1B) The point of greatest spectral overlap of the two light conditions occurred at 241 nm in which the extinction coefficients are 8000 M^−1^ cm^−1^ for VL- and UV-treated photobuforin II. These data indicate that photobuforin II can be isomerized from its *trans* to *cis* isomer.

The photostationary state (PSS) distribution for photobuforin II was also determined by irradiating the peptide in both VL and UV conditions, separating the isomers using HPLC, and integrating under the respective peaks to obtain the distribution of isomers in each state. Baseline separation of the *cis* and *trans* peaks was not achieved and the area under the peak was determined by splitting the peak. This resulted in a PSS distribution of 87.7 % *trans* after irradiation with visible light and 50.6 % *cis* after irradiation with UV light. The PSS distributions were also determined using Shimadzu LabSolutions peak deconvolution software. The PSS distribution determined by deconvolution is 82.6 % *trans* for the VL treated sample and 74.6 % *cis* for the UV treated sample.

### 3.2 Secondary structure of photobuforin II

**Fig. 2:**
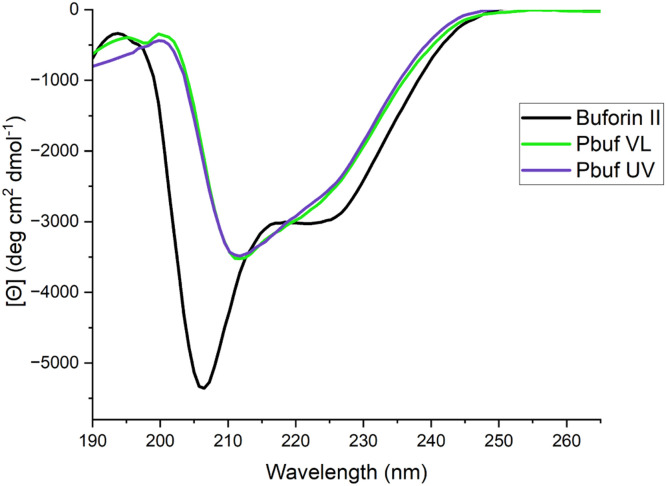
Circular Dichroism spectra of UV and VL treated photobuforin II and buforin II in 50 % TFE in water.

VL and UV treated photobuforin II both exhibited some α-helical structure in 50 % trifluoroethanol:water *(v/v)* (Fig. 2). The percent helicity of photobuforin II was determined and compared to the wildtype peptide using Eq. 1. VL treated photobuforin II is 14.9 % helical and UV treated photobuforin II is 14.7 % helical. The percent helicity is comparable to the wildtype buforin II which was determined to be 15.5 %. [29] From this data it can be inferred that isomerization about the P11Z substitution did not result in the loss of α-helical secondary structure though it does exhibit partial loss of overall secondary structure with respect to the well at 208 nm for buforin II wild type.

### 3.3 Photobuforin II is fluorescent

**Fig. 3:**
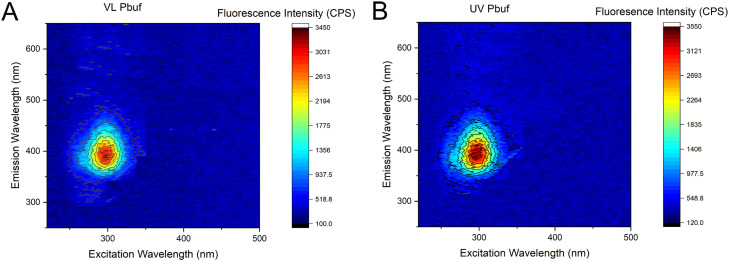
3D Fluorescence spectra of A) VL and B) UV photobuforin II. VL and UV have an emission peak at 390 nm after being excited at 290 nm.

**Fig. 4:**
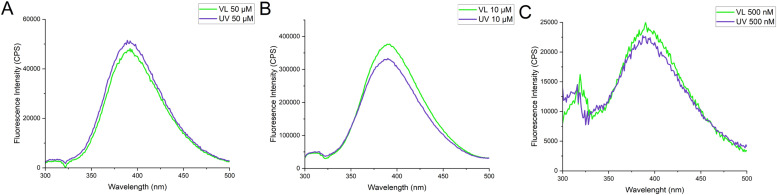
Fluorescence spectra of photobuforin II after incubation in VL and UV light for 10 min. A) 50 µM B) 10 µM C) 500 nM. The fluorometer emission bandwidth for B) and C) was 10 nm whereas the slit size for A) was adjusted to 5 nm due to oversaturation at 10 nm.

DNA binding of buforin II has previously been determined by monitoring the displacement of a fluorescent DNA intercalator, thiazole orange. [30,31] To explore a more direct way to observe binding intrinsic fluorescence of the peptide was explored. The 3D fluorescence spectrum of untreated photobuforin II showed a strong emission peak at 390 nm after being excited at 290 nm giving a Stokes shift of 8840 cm^−1^ (Fig. 3A). The same excitation and emission was observed for UV treated photobuforin II, although it is slightly broader and more intense. Additional 2D scans were performed at 50 µM, 10 µM and 500 nM to determine if the fluorescence was produced by aggregation-induced emission (AIE). VL and UV treated photobuforin II had strong fluorescence emission peaks at as low as 500 nM concentration (Fig. 4). Since photobuforin II is still fluorescent at sub-micromolar concentrations the fluorescence does not appear to be caused by AIE. The large Stoke shift may suggest an intramolecular interaction such as Förster Resonance Energy Transfer (FRET). Photobuforin II contains a phenylalanine residue (F10) that is directly attached to the azobenzene amino acid (Z11). Phenylalanine typically absorbs at 260 nm and emits at 280 nm. Since the max absorbance of photobuforin II is at 290 nm and not 260 nm it does not appear to be exhibiting FRET, unless the absorbance of the phenylalanine has been red shifted.

### 3.4 DNA and vesicle binding of photobuforin II

**Fig. 5:**
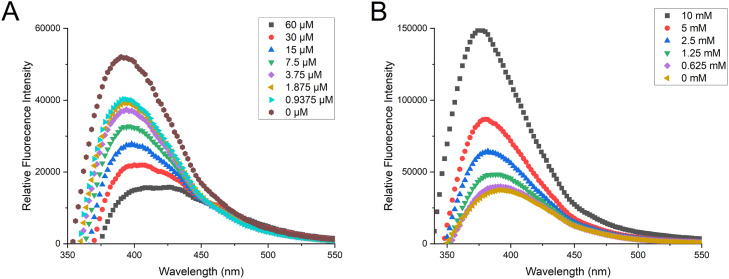
Fluorescence emission spectra of photobuforin II under various conditions. A) Increasing amounts of DNA were added to an aliquot of 15 µM photobuforin II resulting in peptide to DNA ratios of; 1:4, 1:2, 1:1, 2:1, 4:1, 8:1, 16:1. B) Increasing amounts of 3:1 POPC:POPG vesicles were added to an aliquot of 15 µM photobuforin II resulting in peptide to lipid ratios of; 3:2000, 3:1000, 3:500, 3:250, 3:125. All samples were excited at 290 nm.

To determine if photobuforin II was membrane active and binds to DNA like the wildtype peptide the intrinsic fluorescence of the peptide was utilized to detect binding. Binding was determined by monitoring spectral changes in the emission peak after excitation at 290 nm. VL and UV-treated photobuforin II decreased in fluorescence and displayed a red-shift in its emission peak upon binding DNA (Fig. 5A). On the contrary, binding of 3:1 POPC:POPG vesicles resulted in an increase in the emission peak and a blue-shift in the fluorescence (Fig. 5B). The effect of lipid vesicles has been observed to detect binding of tryptophan containing peptides. Tryptophan also increases in fluorescence and undergoes a blue-shift once bound to lipid vesicles. We suggest that this is a new method to determine membrane binding using azobenzene amino acids in addition to tryptophan, tyrosine, or phenylalanine. [32,33] The contrasting spectral changes may be due to the difference in peptide environment in the two scenarios. In the case of DNA the F10 and Z11 residues are likely in a polar charged environment, the basic residues interacting with the phosphate backbone. Upon vesicle binding residues F10 and Z11 appear to be embedded in the hydrophobic inner leaflet of the membrane thus causing an increase in fluorescence intensity. This converse relationship can be useful for future study of the mechanism in which photobuforin II interacts with the cell membrane.

**Fig. 6:**
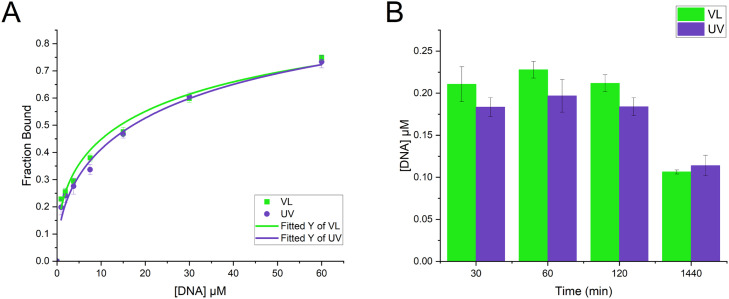
DNA binding of photobuforin II. Relative fluorescence at excitation of 290 nm and emission of 392 nm was converted to fraction bound using Eq. 2. Peptide to DNA ratios: 1:4, 1:2, 1:1, 2:1, 4:1, 8:1, 16:1. (A) Binding curve of photobuforin II with a 120 min incubation. (B) Binding constants *k* over time fit to Eq. (3).

For DNA binding the fluorescence emission at 392 nm was normalized to give the fraction bound using Eq. (2). The average fluorescence of the buffer was used for F_min_ and the fluorescence of the peptide with no DNA was used for F_max_. Fig. 6A shows the binding curve of VL and UV-treated photobuforin II after a 120 min incubation. To determine equilibrium of the system, binding constants were evaluated over time (Fig. 6B). The DNA binding constant was determined to be 0.11 µM (+/- 0.002) for VL-treated photobuforin II and 0.11 µM (+/- 0.011) for UV treated photobuforin II after a 24 h incubation. VL and UV-treated photobuforin II do not exhibit statistically significant differences in DNA binding. Based on the fit to our data we may estimate that the point of 50 % Fraction Bound is roughly between 13 μM for VL and 16 μM for UV conditions. Literature values of 50 % binding for buforin II have determined the value to be around 11 μM. [34] Thus, photobuforin II appeared to maintain much of the wild type peptide affinity for DNA binding. The binding of DNA to buforin II most likely involves electrostatic interactions between the phosphate groups of the DNA and basic residues of the peptide. Since the net charge of photobuforin II is the same as buforin II it is reasonable that DNA binding is retained.

**Fig. 7:**
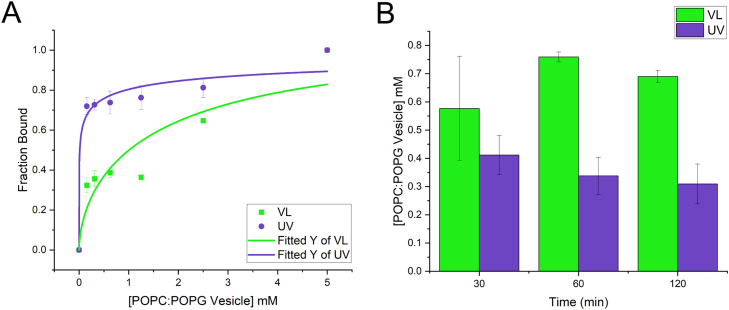
Vesicle binding of photobuforin II. Relative fluorescence at excitation of 290 nm and emission of 380 nm was converted to fraction bound using Eq. 2. Peptide to lipid ratios: 1:2500, 1:1250, 1:625, 4:1250, 4:626, 1:78. (A) Binding curve of photobuforin II after 120 min incubation. (B) Binding constants k over time fit to Eq. 3.

Membrane binding was determined by using the fluorescence emission peak at 380 nm and the fraction bound was determined using Eq. 2. The fluorescence of the buffer was used for F_min_ and the fluorescence at the highest concentration of lipids was used for F_max_. The data was fitted using Eq. (3) to give binding constants. These data appear in Fig. 7. Equilibrium appeared to be reached after 120 min incubation which gave a binding constant of 0.69 mM (+/- 0.021) for VL-treated photobuforin II and 0.31 mM (+/- 0.070) for UV-treated photobuforin II. UV-treated photobuforin II has a 2.2 fold higher binding constant to 3:1 POPC:POPG vesicles compared to VL-treated photobuforin II. It is important to note that while binding is required for translocation of the peptide across the membrane higher binding affinities may result in the loss of the translocation function.

### 3.5 Membrane activity of photobuforin II

**Fig. 8:**
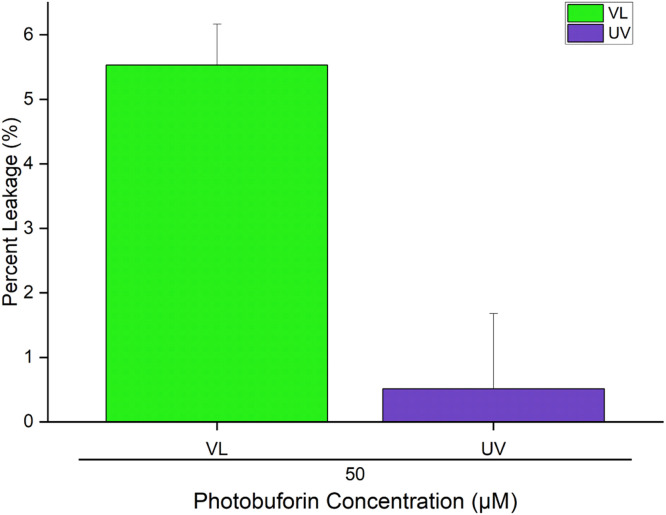
50 µM photobuforin II after 10 min of VL and UV treatment and 24 h incubation with 3:1 POPC:POPG vesicles.

The membrane activity of photobuforin II was tested by vesicle leakage assays which utilize 100 nm vesicles 3:1 POPC: POPG with encapsulated Tb-citrate. The peptide was added to the vesicles in a solution of dipolinic acid (DPA). Upon vesicle leakage citrate is replaced by DPA which forms a fluorescent complex with Tb. Percent leakage was determined by Eq. 4. After a 24 h incubation VL-treated photobuforin II showed 5.5 % leakage (+/- 0.63 %) and UV treated photobuforin II showed 0.51 % leakage (+/- 1.16 %) at a concentration of 50 µM peptide (Fig. 8). At such high peptide to lipid ratios it is safe to say that the P11Z substitution did not result in a change in mechanism from translocation to pore formation as was found with the P11A substitution. [6] It is also interesting that VL-treated photobuforin II induced 11-fold more leakage than UV-treated photobuforin II.

## 4 Conclusions

In a previous work we demonstrated that replacing proline with an azobenzene amino acid in melittin resulted in retention of its alpha helical structure and lytic activity. [14] This work provides further evidence that an azobenzene amino acid can be used to substitute proline in alpha-helical membrane active peptides. The helical structure of the wild-type peptide was at least somewhat preserved, and the overall mechanism of action does not appear to be dramatically altered. Photobuforin II can also be isomerized between its *trans* and *cis* isomers when irradiated by visible and UV light respectively while also gaining the ability to fluoresce. Photobuforin II has good PSS values in water which are necessary for determining differences in the structure-function relationship of the two isomers and for photopharmacological applications. VL-treated photobuforin II has a 2.2-fold lower membrane binding constant compared to UV-treated photobuforin II but caused 11-fold more leakage. The intrinsic fluorescent property of the peptide can be utilized to develop a translocation assay in which DNA is encapsulated in lipid vesicles. The fluorescence of the peptide can be monitored in real time and changes in fluorescence would provide information on the location of the peptide. In theory the fluorescence should increase initially as the peptide binds to the membrane then decrease as it translocates across the membrane into the aqueous space occupied by the DNA. This assay would provide quantitative data with regards to translocation over time. In addition to this minimum inhibitory concentration (MIC) assays can be performed to determine if the peptide has retained its antimicrobial activity. Future work may also explore the possibility of using azobenzene to create other fluorescent peptides by placing it next to aromatic amino acids.

## Funding

Startup funding for this project was provided by Seton Hall University.

## CRediT authorship contribution statement

**Cristina R. Ventura:** Conceptualization, Validation, Formal analysis, Visualization, Writing – original draft. **Gregory R. Wiedman:** Conceptualization, Formal analysis, Project administration, Supervision, Writing – review & editing.

## Declaration of Competing Interest

The authors have no competing interests or conflicts of interest to declare.

## Data Availability

Data will be made available on request.

## Appendix Supplementary materials

Image, application 1
