# Supplementary material for: Photobuforin II, a fluorescent photoswitchable peptide
Source: BBA Adv. 2023 Sep 29;4:100106. doi: 10.1016/j.bbadva.2023.100106 (PMC10568295; doi:10.1016/j.bbadva.2023.100106)
Supplement: Supplementary file 1 [file mmc1.docx]

**
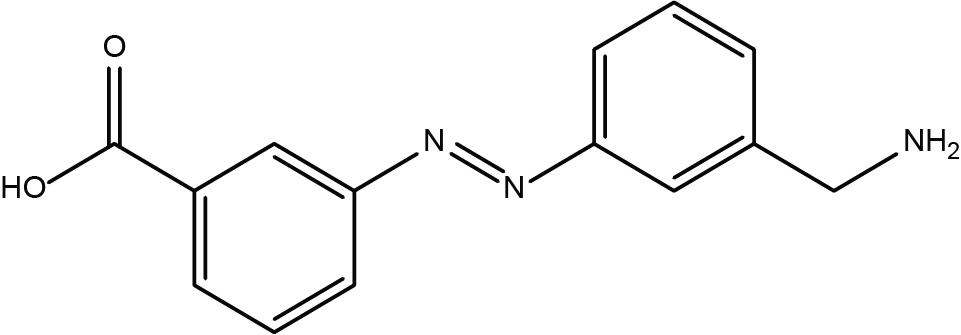
**

**Supplementary 1 Structure of Azobenzene Amino Acid**

**Supplementary 2 ^13^C NMR spectrum of protected azobenzene. Samples were run in the following manner: (126 Mhz, dmso) tert-butyl-2-(3-((3-(((((9H-fluoren-9-yl)methoxy)carbonyl)amino)methyl) phenyl)diazinyl)phenyl)acetate) δ 156.79, 149.07, 144.38, 141.20, 140.76, 129.12, 128.07, 127.54, 125.70, 120.57, 115.03, 113.02, 112.94, 112.08, 112.01, 65.90, 47.25, 44.51.**


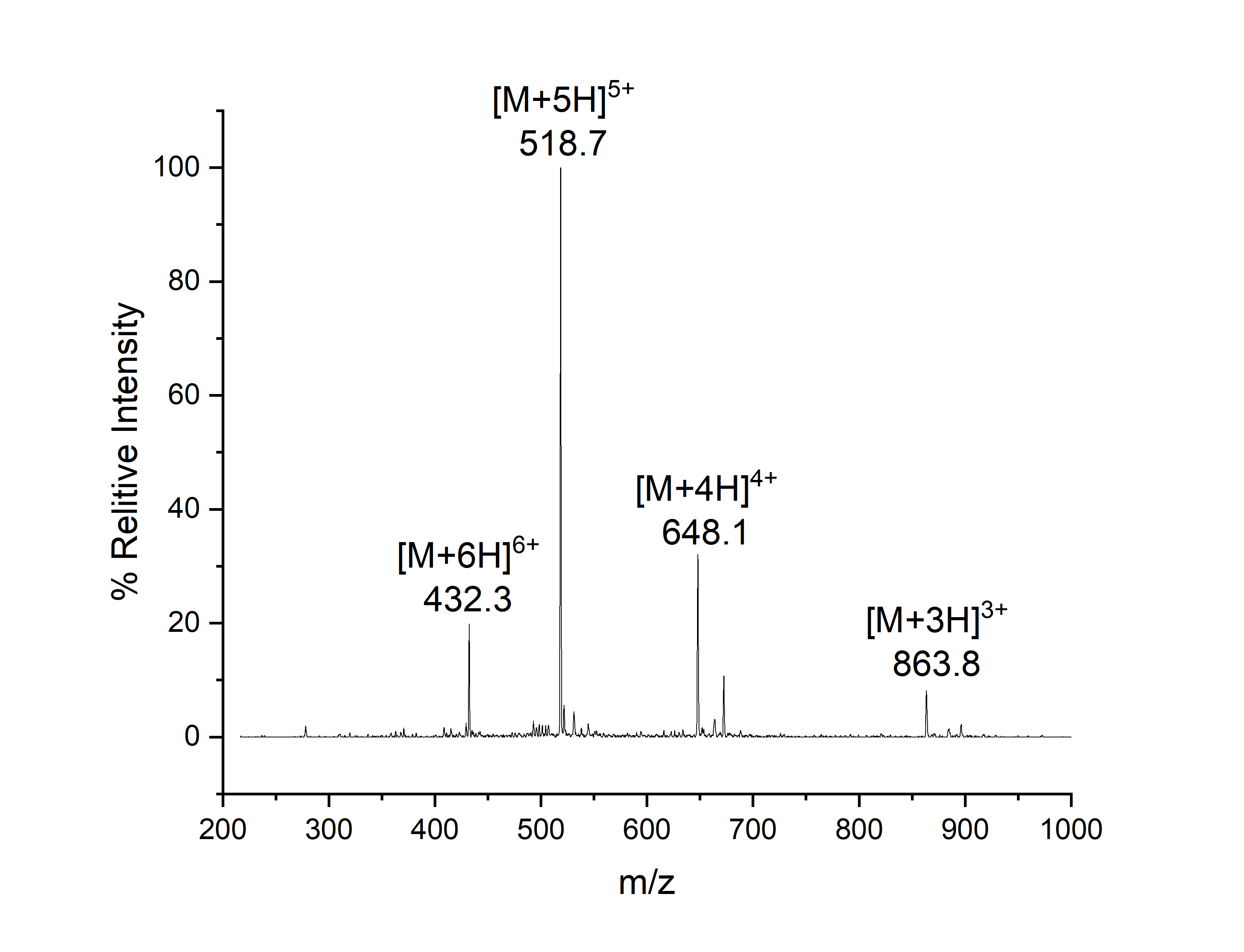


**Supplementary 3** **Mass spectrum of photobuforin II Expected: [M+6H]^6+^ = 432.4, [M+5H]^5+^ = 518.7, [M+4H]^4+^ = 648.1, [M+3H]^3+^ = 863.8.**


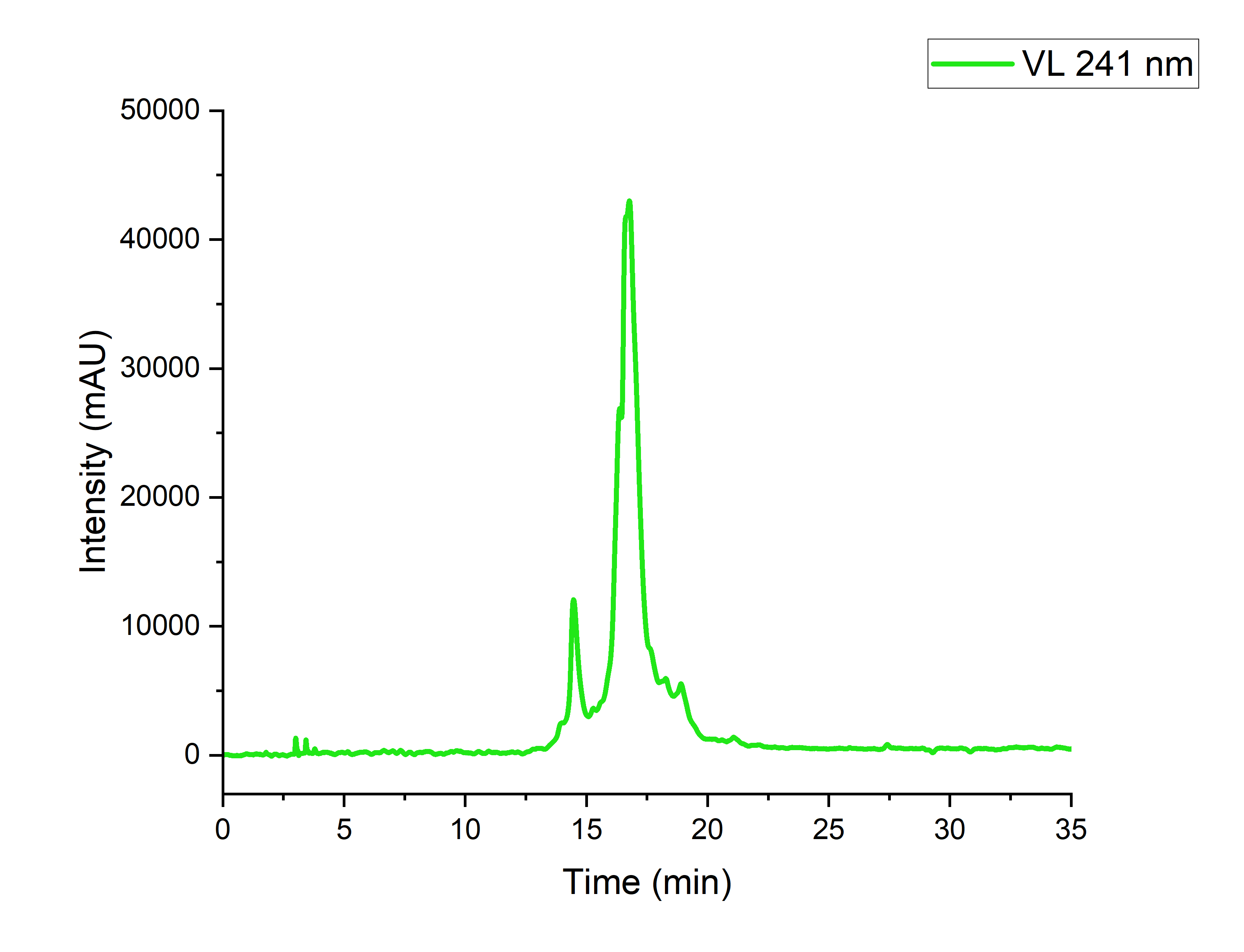


**Supplementary 4 Chromatogram of VL treated photobuforin II at 241 nm. Retention time: 14.472 min cis, 16.401 min trans. Overall estimation was 82.6% trans.**


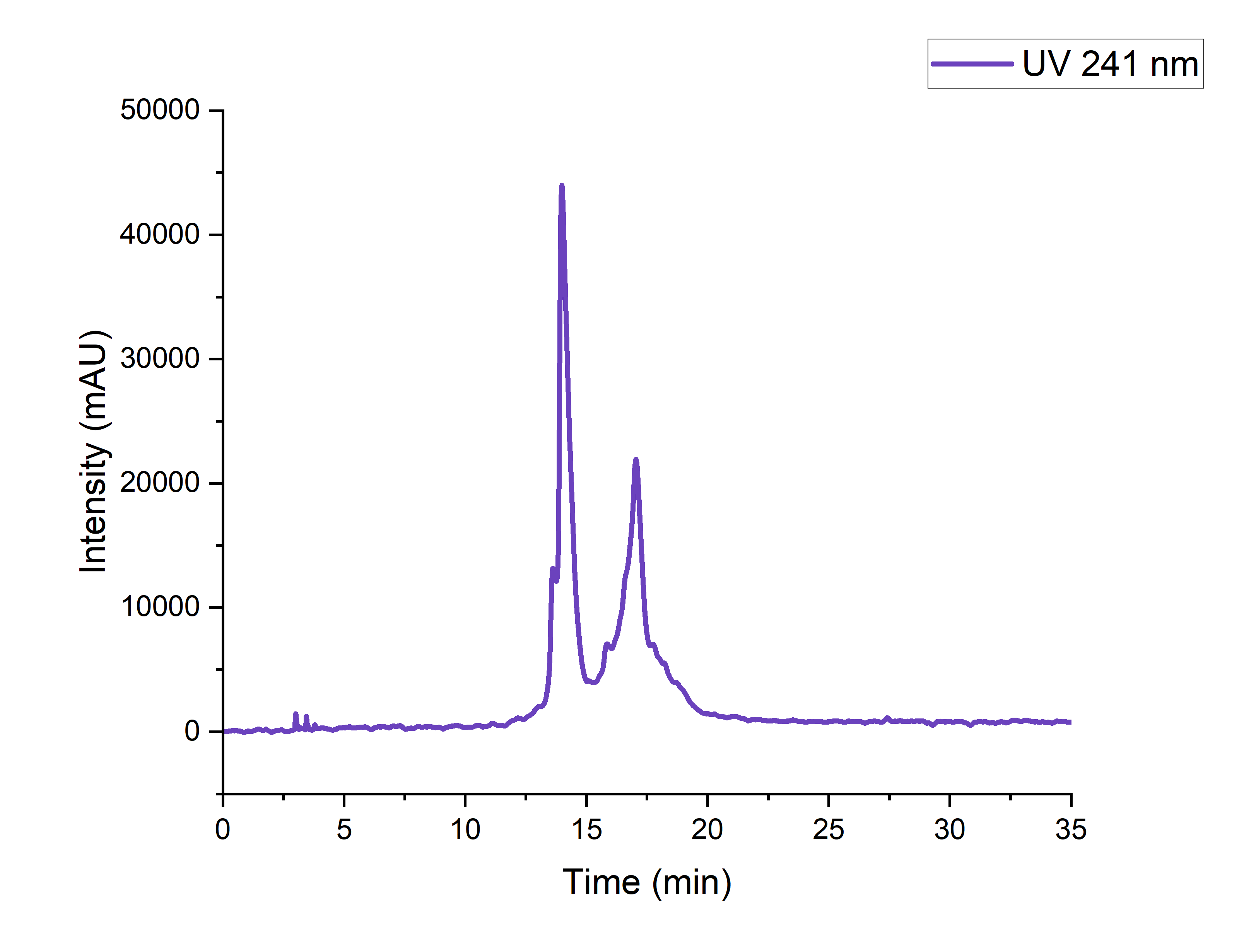


**Supplementary 5 Chromatogram of UV treated photobuforin II at 241 nm. Retention time: 13.986 min cis, 17.042 min trans. Overall estimation was 25.4% trans.**


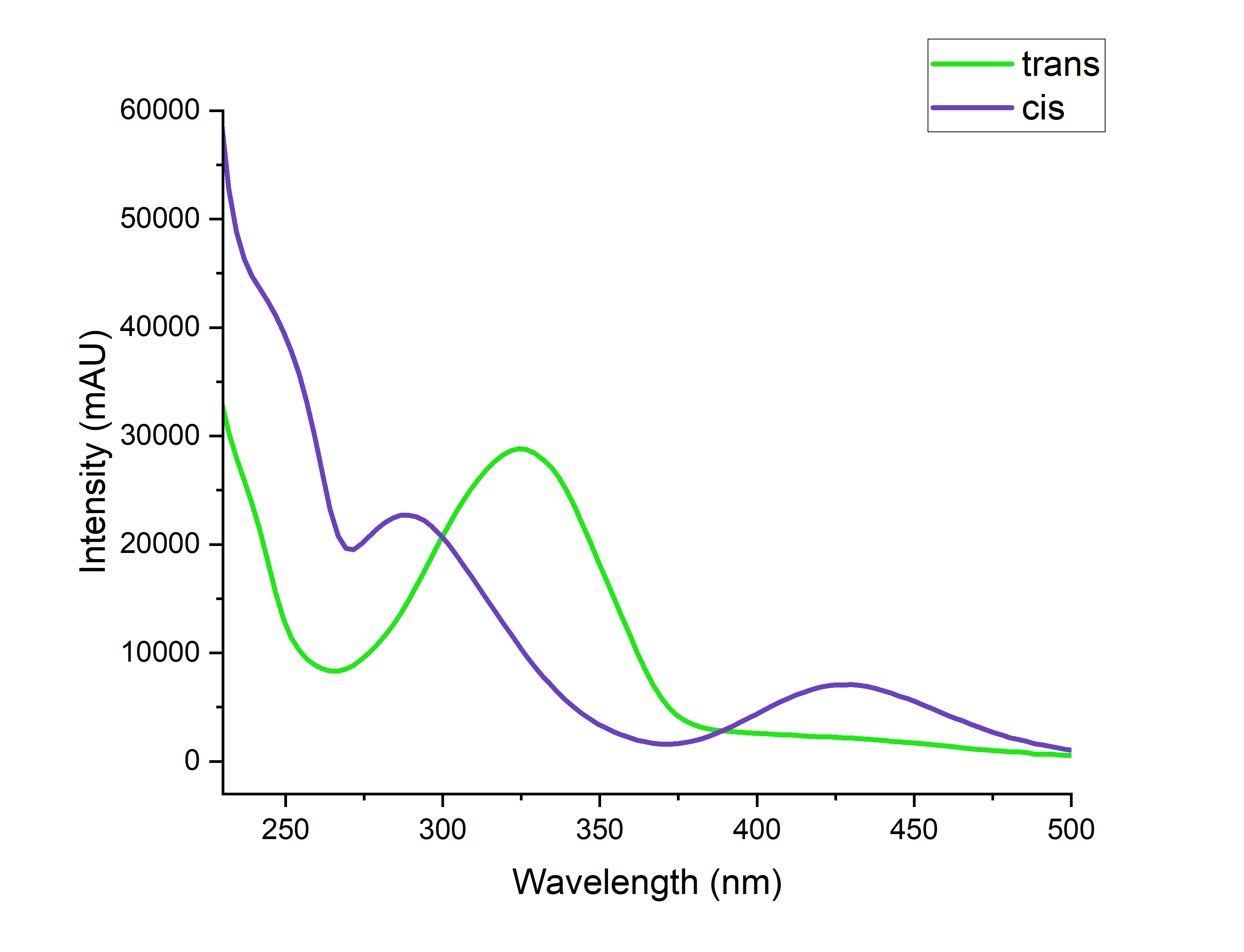


**Supplementary 6 UV photobuforin II PDA slice from 13.986 min (cis), 17.042 min (trans)**


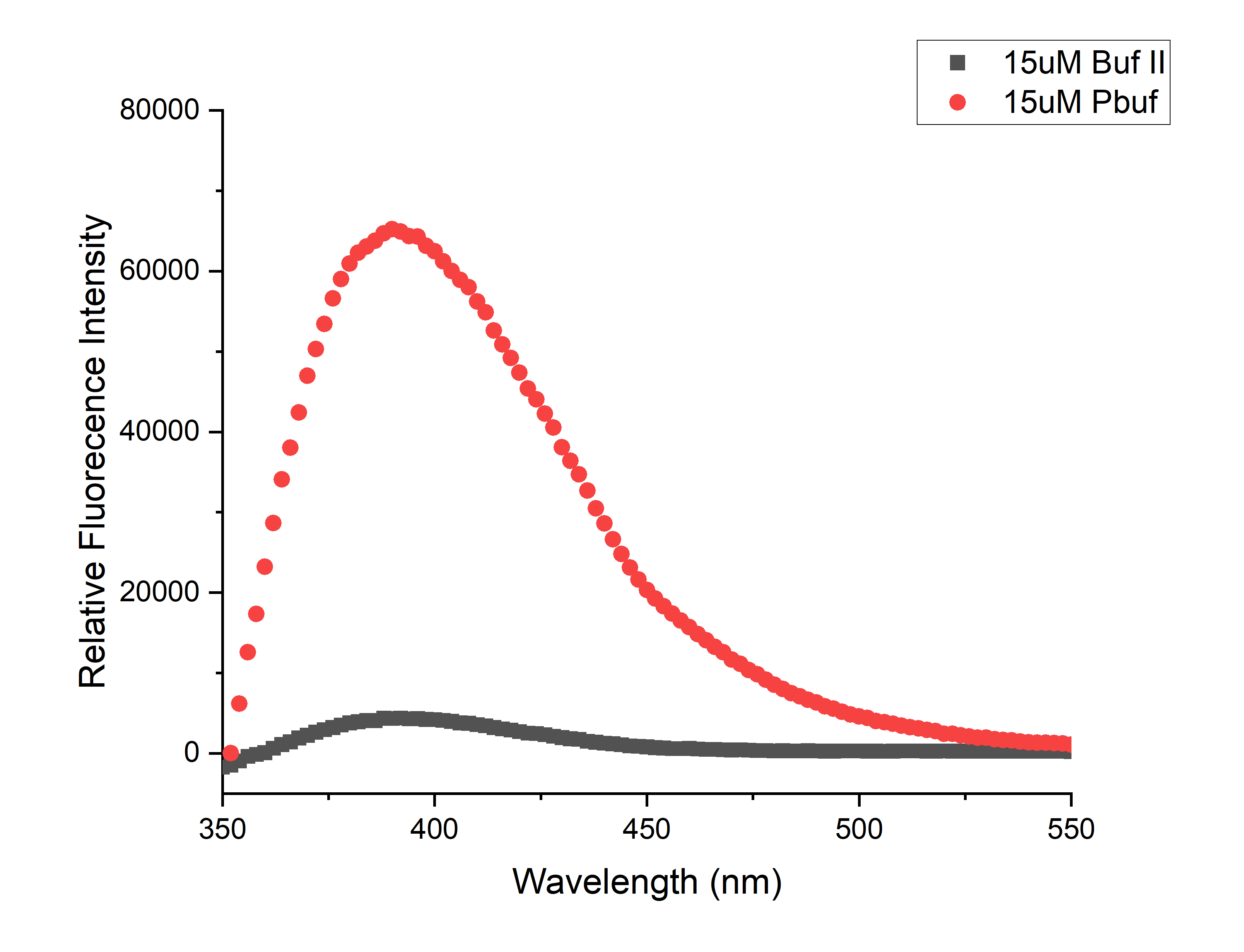


**Supplementary 7 Fluorescence spectrum of 15uM buforin II and photobuforin II. Excitation 290 nm.**
